# Optimizing phosphate fertilizer rate and sources for enhancing yield and grain zinc of maize under saline conditions

**DOI:** 10.3389/fpls.2026.1937429

**Published:** 2026-09-10

**Authors:** Wei Yan, Shuaibing Wu, Zhonghua Wang, Qi Maio, Yinshan Xie, Houhua Liu, Rixin Gao, Lingxiao Zhang, Xiao Sun, Yanfang Xue, Zhenling Cui

**Affiliations:** 1 National Engineering Research Center of Wheat and Maize/Key Laboratory of Biology and Genetic Improvement of Maize in Northern Yellow-Huai Rivers Plain, Ministry of Agriculture and Rural Affairs, Maize Research Institute, Shandong Academy of Agricultural Sciences, Jinan, China; 2 State Key Laboratory of Nutrient Use and Management, College of Resources and Environmental Sciences, China Agricultural University, Beijing, China; 3 Anhui Provincial Key Laboratory of Nutrient Cycling and Arable Land Conservation, Soil and Fertilizer Institute, Anhui Academy of Agricultural Sciences, Hefei, China; 4 College of Agricultural and Biology, Liaocheng University, Liaocheng, Shandong, China

**Keywords:** coastal saline soil, grain zinc biofortification, maize, phosphorus fertilizer management, phosphorus-zinc antagonism

## Abstract

Crops grown on coastal saline-alkali soils in China often receive excessive phosphorus (P) fertilization to overcome low P availability. However, it often leads to reduced grain zinc (Zn) concentration–a critical micronutrient for human and animal health. The interactive effects of P application rates and fertilizer sources on maize (*Zea mays* L.) yield and grain Zn accumulation under saline stress remain unclear. This study aims to identify optimal P fertilizer rates and sources to reduce the P-Zn antagonism while sustaining high yield. A three-year field experiment was conducted to test five P rates (0, 13, 26, 39, and 78 kg P ha^–1^) and three P fertilizer sources including single superphosphate (SSP), monoammonium phosphate (MAP), and ammonium polyphosphate (APP). Measurements included maize yield, grain and straw concentrations of P and micronutrients, soil Olsen-P, DTPA-Zn. Linear-plateau regression and structural equation modeling were used to identify optimal P management. Results showed that a yield plateau of 9.14 Mg ha^−1^ was attained at an optimal P fertilizer rate of 24.1 kg P ha^−1^, which closely matched the treatment level of 26 kg P ha^−1^. At this rate, the three-year average yield was highest with SSP, intermediate with APP, and lowest with MAP. Compared to no P supply, increasing P application rates significantly reduced grain Zn concentrations by 13.1–26.4% and straw Zn concentrations by 27.9–47.2%, even though it increased Zn harvest index. Compared to APP, SSP and MAP resulted in 8.7% and 9.7% of increase in grain Zn concentration, respectively. Structural equation modeling revealed that aboveground biomass, grain yield, soil Olsen-P, and soil pH negatively affected grain Zn concentration. At the yield plateau, grain and straw Zn concentrations were 14.1 and 9.5 mg kg^–1^, respectively, with soil DTPA-Zn around 0.81 mg kg^–1^. These results suggest that applying SSP at 26 kg P ha^–1^ significantly improved maize yield compared with MAP, while also maintaining higher grain Zn concentration compared to APP. The optimizing P rates and sources and rates can partially mitigate P-Zn antagonism while sustaining high maize yields in the coastal saline soil.

## 1 Introduction

Soil salinization and alkalization represent significant challenges to food production security and land use efficiency. Globally, more than 950 million hectares of lands are affected salinity, representing 10% of the earth’s total land surface (Singh, 2021; Xia et al., 2019). In China, saline-alkali lands cover approximately 100 million hectares, with over 80% remaining undeveloped (Xie et al., 2021). Coastal saline-alkali soils account for around 7% of China’s arable land, and are predominantly distributed along the Bohai Rim and the Yellow Sea (Li et al., 2025). These soils have high salinity and alkalinity, low soil organic matter, resulting in poor nutrient availability and low crop productivity with reported reductions from 10% to 90% (Ma et al., 2023; Xie et al., 2021). Improving crop production on coastal saline-alkali soils has become an urgent task for agricultural sustainability and national food security in China.

Maize is a major staple for humans and livestock, with global production reached 1,242 million tons in 2023. China produced 289 million tons in 2023, accounting for 45% of national cereal output and 23.3% of global maize production (FAO, 2026). Among major cereals, maize, rice and wheat are typically low in zinc (Zn) and iron (Fe) concentrations and they constitute nearly two-thirds of dietary energy intake in many developing countries (Saha et al., 2015). The bioavailability of Fe and Zn in cereal grains is also low because of the presence of antinutritional compounds such as phytic acid and phenolic compounds (Thompson, 1993). Previous study reported that approximately 80% of the total P content in seeds exists in form of phytate (Febles et al., 2002), which impairs biological utilization by binding essential minerals like Zn and Fe. Micronutrient deficiencies, particularly Zn and Fe, pose significant challenges not only to human health but also to livestock nutrition. Higher micronutrient concentrations and/or bioavailability in plant-derived food and feed improve both human health and animal welfare (Grujcic et al., 2018). Therefore, enhancing Zn and Fe concentrations and/or bioavailability in maize grains is critical for crop productivity and nutritional value.

Phosphorus (P) is a key limiting nutrient in many agricultural soils, particularly in calcareous and alkaline environments. In these soils, P easily forms insoluble compounds with cations such as calcium (Ca) and magnesium (Mg), leading to low availability (Nadeem et al., 2024; Wei et al., 2021). High salinity and elevated pH can further intensify P fixation, reducing P availability (Ma et al., 2023; Xie et al., 2022). To overcome low P levels, farmers often apply P fertilizer at rates above crop requirements to enhance soil P fertility and increase crop yields (Ma et al., 2025; Hou et al., 2025). However, P use efficiency for maize in China is only 16%, indicating that a significant portion of applied fertilizer either accumulates as legacy P or is lost to the environment (Yan et al., 2022). In saline-alkali soils, high pH combined with excessive P fertilization also intensified Zn deficiency in crops such as maize, wheat, rice, and bean (Ganapati et al., 2022; Nadeem et al., 2024; Nath et al., 2024). Studies on calcareous soils have demonstrated that excessive P fertilization can strongly suppress Zn uptake by crop roots and reduce grain Zn concentration, particularly in maize and wheat due to reduced arbuscular mycorrhizal (AM) colonization (Hui et al., 2022; Liu et al., 2024; Wang et al., 2024; Zhang et al., 2017b). Nevertheless, there remains a notable research gap regarding the interaction between P and Zn, specifically for maize grown under saline-alkali conditions. Because maize is highly sensitive to Zn deficiency (Liu et al., 2017; Wang and Jin, 2007), optimizing P fertilizer management is essential for enhancing maize yield while mitigating any antagonistic effects of excessive P on Zn availability in saline-alkali soils.

A range of commercially available P fertilizers, including single superphosphate (SSP), monoammonium phosphate (MAP), diammonium phosphate (DAP), calcium magnesium phosphate (CMP), and ammonium polyphosphate (APP), vary widely in their effects on soil P availability and crop performance depending on soil types, climatic conditions and management practices. Previous research shows that SSP can perform as well as DAP in enhancing maize yield, while being less costly (Amanullah et al., 2009). DAP had the strongest yield response in neutral soils, while CMP was most effective in acidic soils, with MAP exhibiting the least effect (Zhao et al., 2022). In calcareous soils, no differences in maize grain yield or aboveground P uptake across different sources of P fertilizer (Wang et al., 2024). Our earlier work showed that applying SSP significantly increased wheat yield and aboveground P accumulation more than MAP and APP under optimal P application rate in coastal saline soil (Liu et al., 2022). Furthermore, we found that optimizing P rate with SSP increased maize yield by 8.1% and increased P agronomic efficiency by 32.4%, due to improved balance of salt ions (such as Ca^2+^, Mg^2+^, K^+^ and Na^+^) in the soil-crop system (Wu et al., 2025). However, information remains limited on how different P rates and P fertilizer sources influence micronutrients, especially Zn availability in maize grown on coastal saline soils.

Therefore, we conducted a 3-yr field trail based on a long-term field experiment in Yellow River Delta. The objectives were to determine: (i) how different combinations of P rate and fertilizer source affect maize yield and grain concentrations and bioavailability of micronutrients, particularly Zn; and (ii) whether optimizing the rate and fertilizer source can reduce the antagonistic effects of P on Zn availability in coastal saline-alkali soil conditions. The molar ratios of P to Zn, Fe, manganese (Mn) and copper (Cu) are used as indicators for their bioavailability (Zhang et al., 2017b).

## 2 Materials and methods

### 2.1 Experimental site description

The field trial was conducted from May 2020 to September 2022, as part of a long-term P-fertilization experiment established in 2017 at the Kenli Breeding Station (37°35’ N, 118°35’ E) in Dongying City, Shandong Province, China. The site is located east of the Yellow River Delta and along the southern coast of Bohai Gulf, characterized by a warm continental monsoon climate with a mean annual temperature of 14.1°C and annual precipitation of 584 mm. During the three growing seasons, the daily mean temperatures were 24.6, 25.8 and 25.2°C, and the accumulated growing degree days (base temperature 10°C) were 1998, 1908 and 2010°C, respectively. Total precipitation was 117 mm in 2020, 316 mm in 2021, and 708 mm in 2022.

The soil is classified as saline-alkali soil with the following characteristics of the 0–20 cm soil layer: pH 7.77 (1:2.5 w/v in water), electrical conductivity (EC) 766 μs cm^–1^, total N 0.95 g kg^–1^, Olsen-P 7.06 mg kg^–1^, available potassium 102 mg kg^–1^, water-soluble Ca^2+^ (1:5 w/v in water) 348 mg kg^–1^, water-soluble Na^+^ (1:5 w/v in water) 472 mg kg^–1^, organic carbon 6.82 g kg^–1^, total salt 2.12 g kg^–1^, and the bulk density 1.24 g cm^–3^. Soil DTPA-extractable Zn, Fe, Mn and Cu were 0.73, 13.49, 8.09 and 1.42 mg kg^–1^, respectively.

### 2.2 Experimental design

The experiment followed a split-plot randomized complete block with four replicates. Each block included five main plots, that received different P application rates at 0, 13, 26, 39, and 78 kg P ha^–1^ (designated as P0, P13, P26, P39, and P78, respectively). Each main plot was further divided into three subplots that received different P fertilizer sources of SSP (16% P_2_O_5_), MAP (N-P_2_O_5_-K_2_O:12-52-0) and APP (N-P_2_O_5_-K_2_O:23-44-0).

Each main plot measured 210 m² (15 m × 14 m), and each subplot measured 70 m² (5 m × 14 m). The maize (cv Zhengdan958) was planted at 75,000 plants ha^–1^ with a row spacing of 60 cm. Nitrogen fertilizer was broadcast at 60 kg N ha^–1^ before sowing, and additional 125 kg N ha^–1^ was applied by deep placement (mid-row band) at the six-leaf stage. Urea (46% N) was used to match the same total application N rate. All P and K fertilizers (60 kg K_2_O ha^–1^ as potassium sulphate) were broadcast as basal fertilizer before sowing and incorporated into the 0–20 cm layer by rotary tillage. Maize was grown under rain-fed conditions without observing any weed or pest issues. Detailed information regarding the experimental site, climate conditions, field practices, and fertilizer characteristics were reported previously (Wu et al., 2025).

### 2.3 Sample collection and measurements

#### 2.3.1 Soil sampling and analysis

At crop maturity, three soil cores (20 cm depth) were collected from each plot using a stainless-steel auger, and combined into one composite sample. Samples were air-dried and passed through a 2 mm sieve prior to analysis. Soil pH was measured in a 1:2.5 soil-water suspension using a pH meter. Electrical conductivity (EC) was measured in a 1:5 soil-water suspension using a conductivity meter. Olsen-P was determined through the molybdenum antimony anti-colorimetric-UV spectrophotometry method. Briefly, soil samples were extracted with 0.5 mol NaHCO_3_ L^–1^ at pH 8.5 (180 rpm, 25°C) for 30 min, followed by the addition of 5 mL of molybdenum antimony reagent. P concentrations in the extracts were determined at 660 nm using an ultraviolet spectrophotometer. DTPA-extractable Zn, Fe, Mn and Cu were determined following Lindsay and Norvell (Lindsay and Norvell, 1978).

#### 2.3.2 Plant sampling and measurements

At maturity, three plants were collected from each plot and separated into grain and straw. All samples were washed with tap water and rinsed with deionized water. They were then dried at 105°C for 30 minutes, then oven-dried at 70°C until constant weight. Dried samples were ground with a stainless-steel grinder and then digested in an HNO_3_-H_2_O_2_ acid mixture using a microwave digestion system (CEM Corp., Matthews, NC, United States). Concentrations of P, Zn, Fe, manganese (Mn), copper (Cu), and boron (B) in the digested solutions were determined using an inductively coupled plasma optical emission spectrometry (ICP-OES, Avio™ 200, PerkinElmer, Waltham, MA, United States). Grain yield was determined by harvesting all ears within a central 6 m² area of each plot and is reported at 15.5% moisture. The term “shoot” refers to all above-ground parts including straw and grain.

### 2.4 Statistical analysis

Data were organized using Microsoft Excel 2019 (Microsoft Corporation, Redmond, WA, USA). Results were presented as mean ± standard error (n=4). Before analysis of variance, the normality of model residuals was evaluated using the Shapiro–Wilk test, and the homogeneity of variances was assessed using Levene’s test. Analysis of variance (ANOVA) was conducted using SAS version 9.4 (SAS Institute Inc., Cary, NC, USA) to determine the main and interactive effects of P application rate, fertilizer source, and year on soil micronutrient contents, maize growth indices, and micronutrient contents. When the ANOVA indicated significant treatment effects, treatment means were separated using Fisher’s protected least significant difference (LSD) test at the 5% significance level.

Multiple regression analyses were also performed using SAS version 9.4 to evaluate the relationships among soil properties, maize growth parameters, and nutrient uptake. The coefficients of determination (R²) and associated P values were reported. Pearson’s correlation analysis was conducted using OriginPro, version 2024b (OriginLab Corporation, Northampton, MA, USA), to assess linearrelations among the measured variables. Correlation plots were generated to visualize significant relationships (p < 0.05).

Principal component analysis (PCA) was performed in R version 4.3.2 (R Foundation for Statistical Computing, Vienna, Austria) using the “prcomp” function in the “stats” package to identify the principal variables contributing to variation in soil and crop micronutrient concentrations across fertilizer P application rates, fertilizer sources, and years. PCA results were visualized using the “factoextra” package. All R analyses were conducted in RStudio version 2026.01.0 + 392, “Apple Blossom” Release (Posit Software, PBC, Boston, MA, USA), running on Microsoft Windows. Partial least squares structural equation modeling (PLS-SEM) was performed using SmartPLS version 4.1.0.3 (SmartPLS GmbH, Bönningstedt, Germany) to investigate the direct and indirect pathways through which P fertilizer sources and application rates influenced maize growth and nutrient uptake. PLS-SEM was selected because of its suitability for relatively small sample sizes, complex model structures, and data that may not satisfy multivariate normality assumptions. Path coefficients with p < 0.05 were considered statistically significant, whereas those with 0.05 ≤ p < 0.10 were considered marginally significant. Model fit was evaluated using the standardized root mean square residual (SRMR) and the normed fit index (NFI).

## 3 Results

### 3.1 Maize yields

**Figure 1:**
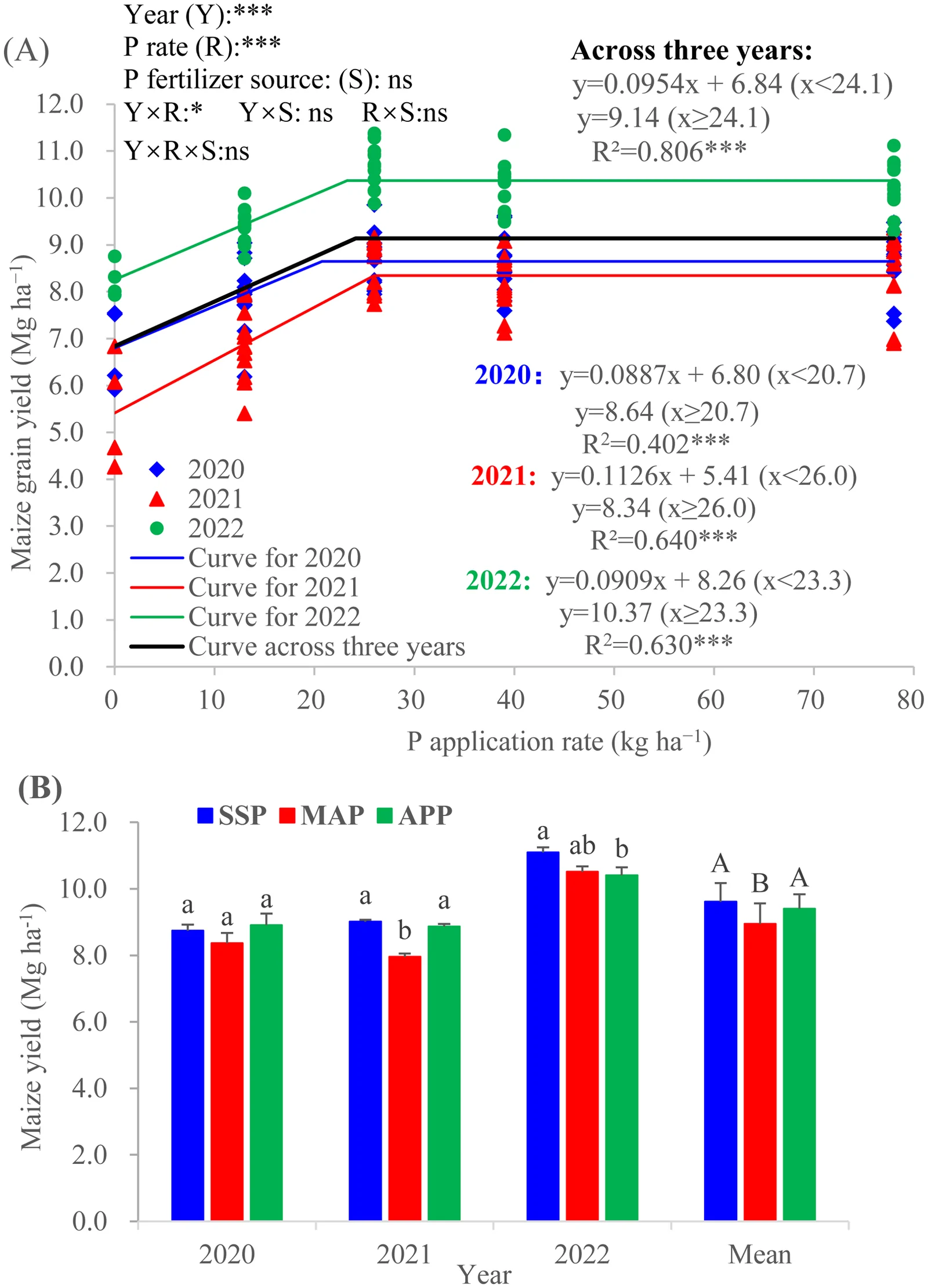
Response of maize yield to different P application rates in 2020, 2021 and 2022 **(A)**, and maize yield under the P26 treatment (26 kg P ha^–1^) with different P fertilizer sources across the three years **(B)**. Results are means of four replicates. Different uppercase and lowercase letters indicate significant differences among fertilizer P sources in **(B)**. *significant at *P* < 0.05, ***significant at *P* < 0.001, ns, not significant; SSP, single superphosphate; MAP, monoammonium phosphate; APP, ammonium polyphosphate.

Grain yield was significantly influenced by P fertilizer rate and cropping year, and their double interactions, but not by fertilizer source or most interactions (**Figure 1A**). In all three years, maize grain yield initially increased with P rate and then reached a plateau. The linear-plateau model estimated yield plateaus of 8.64, 8.34, and 10.37 Mg ha^−1^ at threshold P rates of 20.7, 26.0, and 23.3 kg P ha^−1^ in 2020, 2021, and 2022, respectively. Across the three years, a yield plateau of 9.14 Mg ha^−1^ was attained at an optimal P fertilizer rate of 24.1 kg P ha^−1^, which closely matched the P26 treatment (26 kg P ha^−1^) (**Figure 1A**). Under this rate, the three-year average yield was highest with SSP, intermediate with APP, and lowest with MAP (**Figure 1B**).

### 3.2 Nutrient concentrations in grain and straw

Fertilizer P application rates significantly affected concentrations of Zn, Fe, and Cu in grain, but had no effect on Mn or B (**Table 1**). In straw, P application rates significantly affected the concentrations of Zn, Mn, and B concentrations but not Fe and Cu. Grain Zn concentration decreased as P rate increased from P0 to P78 (**Table 1**). Grain Fe and Cu also decreased from P0 to P26 but showed no further changes with additional P application. In straw, Zn and B concentrations gradually declined as the P rate increased. Straw Mn concentration decreased from P0 to P13, and then remained stable with additional P application.

**Table 1:** Micronutrient concentrations in maize grain and straw as affected by P rates, P sources, and cropping years.

Treatment	Grain micronutrient concentration (mg kg^–1^)					Straw micronutrient concentration (mg kg^–1^)				
	Zn	Fe	Mn	Cu	B	Zn	Fe	Mn	Cu	B
P rate (R)										
P0	17.24a	18.20a	3.22a	1.46a	1.59a	14.82a	199.98a	29.12a	5.14a	7.12a
P13	14.99b	17.61ab	3.22a	1.34b	1.47a	10.69b	211.08a	26.69bc	5.35a	6.72b
P26	13.69c	17.27b	3.19a	1.24c	1.46a	9.48c	211.48a	28.04ab	5.15a	6.69b
P39	13.73c	16.79b	3.34a	1.24bc	1.55a	8.50cd	198.67a	25.20c	5.29a	6.54bc
P78	12.68d	17.61ab	3.42a	1.15c	1.51a	7.82d	220.56a	26.24bc	5.21a	6.32c
P source (S)										
SSP	14.10a	17.45a	3.31a	1.26a	1.48a	9.23a	205.64a	27.37a	5.42a	6.59a
MAP	14.24a	17.58a	3.27a	1.27a	1.52a	9.06a	206.33a	26.30a	5.04a	6.49a
APP	12.97b	16.92a	3.30a	1.20a	1.48a	9.07a	219.37a	25.97a	5.30a	6.63a
Year (Y)										
2020	14.82a	16.82b	3.62a	1.55a	1.73a	7.58b	191.76b	23.88c	5.79a	5.29c
2021	13.73b	16.52b	3.75a	1.04c	1.66a	6.87c	162.42c	30.73a	5.66a	6.05b
2022	13.57b	18.82a	2.48a	1.18b	1.11b	14.24a	274.74a	25.61b	4.28b	8.50a
Source of variation										
Y	<0.0001	<0.0001	ns	<0.0001	<0.0001	<0.0001	<0.0001	<0.0001	<0.0001	<0.0001
R	<0.0001	0.0335	ns	<0.0001	ns	<0.0001	ns	0.0011	ns	<0.0001
S	0.0004	ns	ns	ns	ns	ns	ns	ns	ns	ns
Y*R	ns	ns	ns	0.0023	ns	ns	ns	ns	0.004	0.032
Y*S	ns	ns	ns	ns	ns	ns	ns	ns	ns	ns
R*S	ns	ns	ns	ns	ns	ns	ns	ns	ns	ns
Y*R*S	ns	ns	ns	ns	ns	ns	ns	ns	ns	ns

Fertilizer P sources include superphosphate (SSP), monoammonium phosphate (MAP), and ammonium polyphosphate (APP). Values represent the means of four replicates. Within a column, different letters denote significant difference at *P* < 0.05. ns indicates not significant.

**Figure 2:**
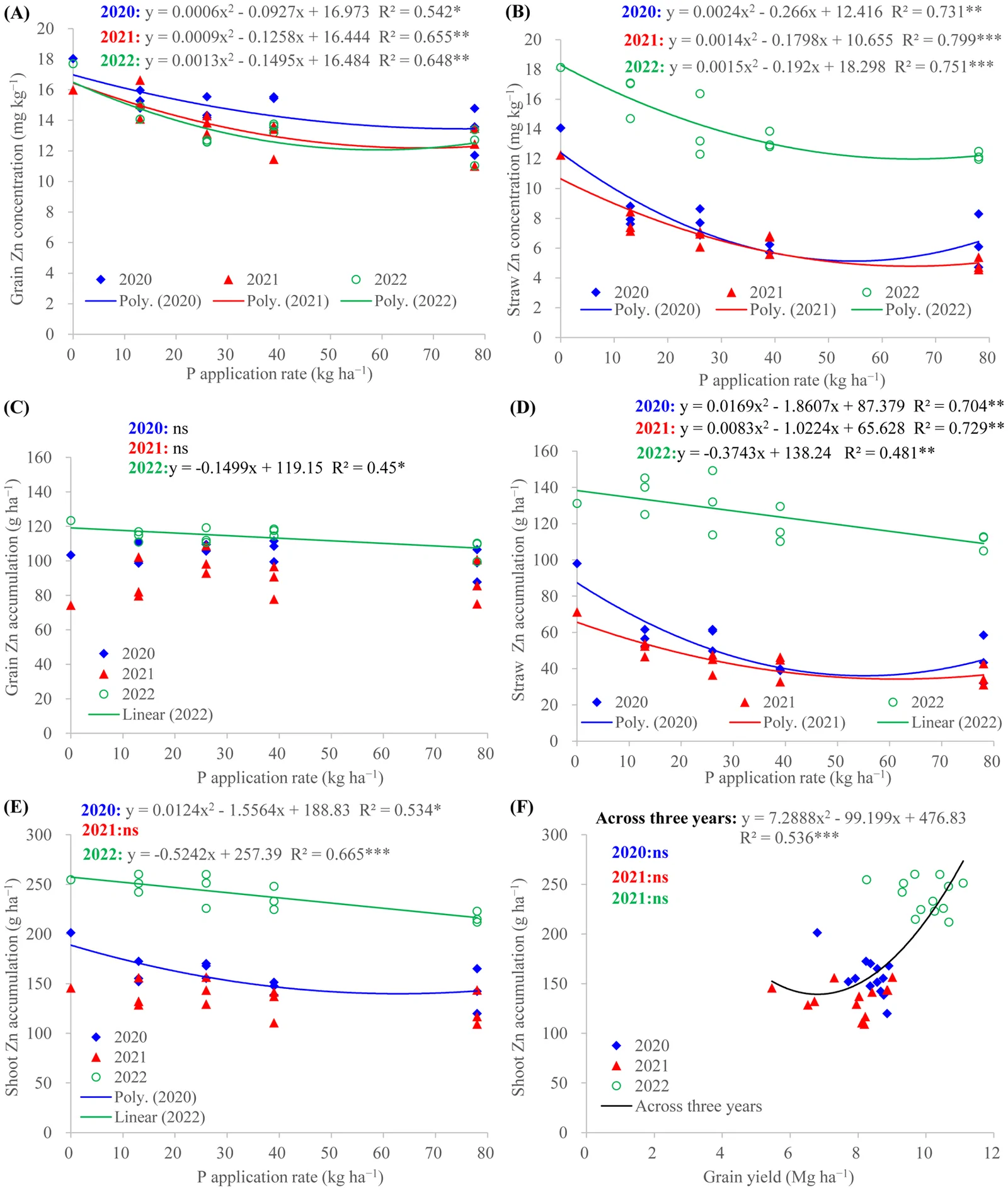
Response of grain Zn concentration **(A)**, straw Zn concentration **(B)**, grain Zn accumulation **(C)**, straw Zn accumulation **(D)**, aboveground Zn accumulation **(E)** to P application rates, and response of aboveground Zn accumulation to grain yield **(F)** in 2020, 2021 and 2022. *significant at P < 0.05, **significant at P < 0.01, ***significant at P < 0.001.

Regression analysis confirmed a consistent quadratic decline in Zn concentration in both grain and straw with increasing P application rates, irrespective of cropping years (**Figures 2A, B**). The reduction in straw Zn was more pronounced than that in grain, leading to a rising grain-to-straw Zn ratio as P rates increased from P0 to P78 (**Supplementary Figure 1**). Fertilizer P sources affected only grain Zn concentration, with APP resulted in lower concentration than SSP or MAP (**Table 1**).

Micronutrient concentrations in both grain (excluding grain Mn) and straw were significantly affected by cropping years. As shown in **Table 1**, grain concentrations of Zn, Cu, and B were highest in 2020, while grain Fe was highest in 2022. Straw concentrations of Zn, Fe, and B were highest in 2022, while straw Mn was highest in 2021, and straw Cu was highest in 2020. Interactions among fertilizer P rates, fertilizer P sources, and cropping years did not affect micronutrient concentrations in both grain and straw, except for a significant effect of the interaction between cropping years and fertilizer P rates on the Cu concentration in both grain and straw as well as B concentration in straw (**Table 1**).

### 3.3 Nutrient accumulation in grain, straw and aboveground

Aboveground accumulation of Zn, Fe, Mn, Cu, and B was significantly affected by P application rates and cropping years, but not by fertilizer P sources or their interactions, with the exception of a notable interaction between cropping years and fertilizer P rates affecting aboveground accumulation of Fe, Cu, and B (**Table 2**). Increasing P application rate from P0 to P39 decreased straw Zn accumulation by 33.8%, while grain Zn accumulation did not change. This led to 15.1% decline in aboveground Zn accumulation. Further increasing P application rate from P39 to P78 caused only a slight decrease in aboveground Zn accumulation (5.6%), even though grain Zn accumulation decreased by 6.5%. Overall, regression analysis confirmed that higher P rates reduced Zn accumulation more strongly in straw than in grain. As a result, the aboveground Zn accumulation declined with quadratically (2020) or linearly (in 2022) as P rates increased (**Figures 2C–E**). Additionally, aboveground Zn accumulation also increased quadratically with grain yield over three years (**Figure 2F**). Mn and B accumulation in grain, straw, and aboveground increased steadily from P0 to P26, and slightly decreased with higher P rates (**Table 2**). Aboveground Fe and Cu accumulation increased significantly with P application rate from P0 to P13, but did not respond with additional P inputs (**Table 2**).

**Table 2:** Micronutrient accumulation in maize grain, straw and aboveground and harvest index as affected by fertilizer P rates, P sources, and cropping years.

Treatment	Grain micronutrient accumulation (g ha^–1^)					Straw micronutrient accumulation (g ha^–1^)					Shoot micronutrient accumulation (g ha^–1^)					Micronutrient harvest index (%)				
	Zn	Fe	Mn	Cu	B	Zn	Fe	Mn	Cu	B	Zn	Fe	Mn	Cu	B	Zn	Fe	Mn	Cu	B
P rate (R)																				
P0	100.4bc	106.9d	18.0c	8.5c	9.0b	100.1a	1342.8c	192.3b	33.7b	47.7b	200.6a	1449.7c	210.3b	42.2b	56.7c	50.9d	7.8a	8.9d	20.5a	16.4c
P13	101.8abc	121.0c	21.4b	9.2abc	9.9b	81.6b	1594.6ab	196.5ab	38.9a	50.6ab	183.4b	1715.6ab	217.9b	48.2a	60.5bc	58.0c	7.8a	10.3c	19.5a	18.0bc
P26	107.1a	137.1a	24.6a	9.7a	11.2a	77.4b	1685.2ab	213.6a	39.1a	53.2a	184.5b	1822.2ab	238.3a	48.8a	64.4a	61.0b	8.6a	10.9bc	20.6a	19.8ab
P39	104.0ab	128.3bc	24.9a	9.4ab	11.5a	66.3c	1523.9bc	185.0b	38.1a	49.6ab	170.3c	1652.2b	210.0b	47.5a	61.1ab	63.9a	8.8a	12.6a	20.3a	20.9a
P78	97.2c	135.6ab	25.8a	8.7bc	11.3a	63.6c	1756.9a	203.8ab	40.1a	50.5ab	160.8c	1892.5a	229.6ab	48.9a	61.8ab	63.2ab	8.0a	11.9ab	18.3a	20.0ab
P types (T)																				
SSP	105.9a	132.8a	24.6a	9.5a	11.0a	73.5a	1627.2a	209.3a	40.7a	51.8a	179.5a	1760.0a	233.9a	50.2a	62.8a	61.6a	8.4a	11.1a	19.3a	19.4a
MAP	104.3a	130.2a	23.6a	9.4ab	11.0a	70.5a	1577.0a	192.7a	36.5a	49.3a	174.8a	1707.2a	216.3a	45.9b	60.3a	62.3a	8.8a	11.7a	21.0a	20.3a
APP	97.3b	128.5a	24.4a	9.0a	10.9a	72.7a	1716.2a	197.2a	39.9a	51.9a	170.0a	1844.6a	221.6a	48.9ab	62.8a	60.7a	7.7a	11.5a	18.8a	19.3a
Year (Y)																				
2020	103.6b	118.4b	25.4a	11.0a	12.2a	53.3b	1333.1b	166.2c	40.2a	37.0b	156.9b	1451.5b	191.6b	51.2a	49.1b	67.4a	8.7a	13.9a	22.1a	25.5a
2021	89.7c	109.0c	24.7b	6.8c	11.0b	45.1c	1089.1c	205.4b	38.0b	40.3b	134.7c	1198.1c	230.1a	44.8b	51.3b	66.8a	9.8a	11.0b	16.0b	21.7b
2022	113.8a	158.6a	21.0c	9.9b	9.3c	124.8a	2429.6a	225.9a	37.7b	74.9a	238.5a	2588.2a	246.9a	47.6b	84.2a	47.9b	6.2b	8.80c	21.2a	11.1c
Source of variation																				
Y	<0.0001	<0.0001	<0.0001	<0.0001	<0.0001	<0.0001	<0.0001	<0.0001	0.021	<0.0001	<0.0001	<0.0001	<0.0001	<0.0001	<0.0001	<0.0001	<0.0001	<0.0001	<0.0001	<0.0001
R	0.0141	<0.0001	<0.0001	0.041	<0.0001	<0.0001	0.0161	ns	0.0244	ns	<0.0001	<0.0001	0.021	0.007	0.0043	<0.0001	ns	<0.0001	ns	0.0093
T	0.001	ns	ns	ns	ns	ns	ns	ns	ns	ns	ns	ns	ns	ns	ns	ns	ns	ns	ns	ns
Y*R	0.006	ns	0.047	0.001	0.002	0.006	0.016	ns	0.011	0.009	ns	0.021	ns	0.013	0.038	<0.0001	ns	ns	0.001	0.021
Y*T	ns	ns	ns	ns	ns	ns	ns	ns	ns	ns	ns	ns	ns	ns	ns	ns	ns	ns	ns	ns
R*T	ns	ns	ns	ns	ns	ns	ns	ns	ns	ns	ns	ns	ns	ns	ns	ns	ns	ns	ns	ns
Y*R*T	ns	ns	ns	ns	ns	ns	ns	ns	ns	ns	ns	ns	ns	ns	ns	ns	ns	ns	ns	ns

Fertilizer P sources include superphosphate (SSP), monoammonium phosphate (MAP), and ammonium polyphosphate (APP). Values represent the means of four replicates. Within a column, different letters denote significant difference at *P* < 0.05. ns indicates not significant.

**Figure 3:**
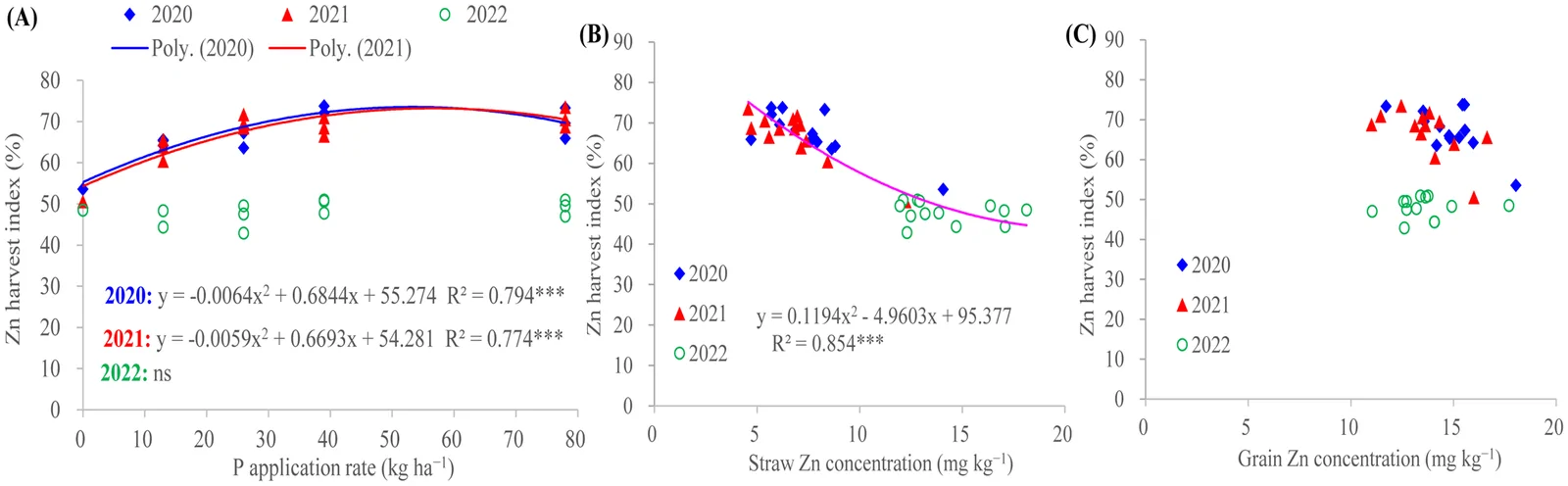
Response of Zn harvest index to P application rates **(A)**, straw Zn concentration **(B)** and grain Zn concentration **(C)** in 2020, 2021 and 2022. ***significant at P <0.001; ns, not significant.

The harvest indices for Zn, Mn, and B were significantly affected by cropping years and P application rates but not fertilizer P types. A progressive increase in the micronutrient harvest indices for Zn, Mn, and B was observed with improving P application rates from P0 to P39, but did not respond with additional P inputs (**Table 2**). Regression analysis showed that Zn harvest index increased quadratically with the increasing P application rates in 2020 and 2021, but this trend was not evident in 2022 (**Figure 3A**). Conversely, a quadratic decrease in the Zn harvest index was noted with the increasing straw Zn concentrations (**Figure 3B**). There was not a significant relationship between Zn harvest index and grain Zn concentration (**Figure 3C**). Therefore, the notably lower Zn harvest index recorded in 2022 than in 2020 and 2021 can be attributed to the significantly higher straw Zn concentrations observed during that year (**Tables 1**, **2**; **Figure 3**). The nutrient harvest indices for Fe and Cu were unaffected by P application rates.

### 3.4 Molar ratios of P to micronutrients in grain and straw

Molar ratios of P/Zn, P/Fe, P/Mn, and P/Cu in grain and straw were generally affected by P application rates, P sources (with the exception of P/Mn in grain and P/Cu in straw), and cropping years (with the exception of P/Zn in grain) (**Table 3**). As P application rates increased from P0 to P78, P concentrations in both grain and straw increased gradually (**Supplementary Table 1**). This increase was accompanied by higher P/Zn, P/Fe, and P/Cu molar ratios in grain, and by higher P/Zn and P/Cu in straw (**Table 3**). The P/Mn ratio in grain increased from P0 to P26, but did not change with additional P application. Similarly, P/Fe and P/Mn ratios in straw increased from P0 to P39, but did not respond at higher P rates. MAP treatment resulted in lower P/Zn, P/Fe, and P/Cu ratios in grain, and lower P/Zn, P/Fe, and P/Mn ratios in straw, compared to the other two P fertilizer sources (**Table 3**).

**Table 3:** The molar ratios of P/Zn, P/Fe, P/Mn, and P/Cu in grain and straw of maize as affected by fertilizer P rates, P sources, and cropping years.

Treatment	Molar ratio of P to micronutrient in grain				Molar ratio of P to micronutrient in straw			
	P/Zn	P/Fe	P/Mn	P/Cu	P/Zn	P/Fe	P/Mn	P/Cu
P rate (R)								
P0	190.1d	156.5d	898.3c	2255.5d	53.3e	3.5d	21.5c	144.9d
P13	275.8c	204.8c	1129.2b	3197.8c	96.8d	4.0dc	28.8b	170.7c
P26	315.6b	216.0bc	1208.6ab	3724.1b	118.6c	4.4bc	30.7b	198.6b
P39	326.5b	227.6b	1166.4b	3702.9b	151.2b	5.1a	37.3a	208.3b
P78	401.9a	245.5a	1284.7a	4678.0a	180.8a	5.0ab	38.8a	230.8a
P source (S)								
SSP	335.4a	232.7a	1244.4a	3905.1ab	140.5a	5.1a	35.5a	211.7a
MAP	299.9b	208.1b	1159.1a	3528.6b	124.5b	4.5b	32.2b	197.9a
APP	354.5a	229.7a	1188.2a	4043.4a	145.6a	4.4b	34.0ab	196.7a
Year (Y)								
2020	310.9a	230.1a	1071.0b	2899.2c	158.1a	4.9b	37.8a	180.9b
2021	323.7a	227.0a	982.9c	4264.3a	163.1a	5.8a	28.2c	181.7b
2022	322.9a	197.9b	1468.8a	3951.3b	70.1b	3.1c	32.9b	230.5a
Source of variation								
Y	ns	<0.0001	<0.0001	<0.0001	<0.0001	<0.0001	<0.0001	<0.0001
R	<0.0001	<0.0001	<0.0001	<0.0001	<0.0001	<0.0001	<0.0001	<0.0001
S	<0.0001	<0.0001	ns	0.0239	0.0073	0.0105	0.0459	ns
Y*R	ns	ns	ns	0.0155	<0.0001	ns	0.0086	0.037
Y*S	0.0075	ns	ns	ns	0.0177	ns	ns	0.0138
R*S	0.0005	ns	ns	ns	ns	ns	ns	ns
Y*R*S	ns	ns	ns	ns	ns	ns	ns	ns

Fertilizer P sources include superphosphate (SSP), monoammonium phosphate (MAP), and ammonium polyphosphate (APP). Values represent the means of four replicates. Within a column, different letters denote significant difference at *P* < 0.05. ns indicates not significant.

### 3.5 Soil pH values, Olsen-P concentration and DTPA-extractable concentrations of Zn, Fe, Mn, and Cu at 0–20 cm depth

Soil EC values, Olsen-P, and DTPA-extractable Zn, Fe, Mn, and Cu differed between years while soil pH remained similar. P application rates had significant effects on these soil parameters, except for DTPA-extractable Mn. Fertilizer P sources affected soil pH values, EC values, and soil Olsen-P concentration (**Table 4**).

**Table 4:** Soil pH and EC values, Olsen-P and soil DTPA micronutrient concentrations at 0–20 cm depth as affected by fertilizer P rates, P sources, and cropping years.

Treatment	pH	EC	Olsen-P	DTPA-Zn	DTPA-Fe	DTPA-Mn	DTPA-Cu
		(μs cm^–1^)	(mg kg^–1^)	(mg kg^–1^)	(mg kg^–1^)	(mg kg^–1^)	(mg kg^–1^)
P rate (R)							
P0	8.37a	245.4b	2.8e	0.72b	16.52c	14.27a	1.76b
P13	8.31b	248.6b	8.6d	0.76b	17.01c	14.18a	1.80b
P26	8.27bc	287.8ab	16.3c	0.77b	16.95c	14.80a	1.78b
P39	8.23c	327.4a	27.3b	0.91a	18.43b	14.87a	1.79b
P78	8.14d	338.1a	52.0a	1.00a	20.94a	15.36a	1.99a
P source (S)							
SSP	8.17b	346.8a	25.4b	0.83a	18.23a	15.03a	1.86a
MAP	8.25a	289.7b	22.7b	0.90a	18.21a	14.46a	1.80a
APP	8.29a	264.9b	30.0a	0.85a	18.56a	14.93a	1.85a
Year (Y)							
2020	8.23a	204.9b	22.2b	0.80b	18.69b	16.06b	1.95a
2021	8.26a	331.2a	23.7b	1.15a	21.62a	19.55a	2.01a
2022	8.26a	352.6a	26.9a	0.59c	14.28c	8.68c	1.53b
Source of variation							
Y	ns	<0.0001	0.0024	<0.0001	<0.0001	<0.0001	<0.0001
R	<0.0001	0.0018	<0.0001	<0.0001	<0.0001	ns	0.0014
S	0.0001	0.0068	<0.0001	ns	ns	ns	ns
Y*R	ns	ns	0.0082	ns	0.0009	ns	0.0169
Y*S	ns	ns	ns	ns	ns	ns	ns
R*S	ns	ns	0.0004	ns	ns	ns	ns
Y*R*S	ns	ns	ns	ns	ns	ns	ns

Fertilizer P sources include superphosphate (SSP), monoammonium phosphate (MAP), and ammonium polyphosphate (APP). Values represent the means of four replicates. Within a column, different letters denote significant difference at *P* < 0.05. ns indicates not significant.

Soil pH declined gradually as P application rate increased from P0 to P78, with a total decrease of 0.23 units. In contrast, soil Olsen-P increased with increasing P application rates from P0 to P78, and EC showed the same pattern (**Table 4**). DTPA-extractable Zn remained stable from P0 to P26, then increased significantly with further P supply. Similar trends were observed for soil DTPA- Fe and Cu (**Table 4**).

**Figure 4:**
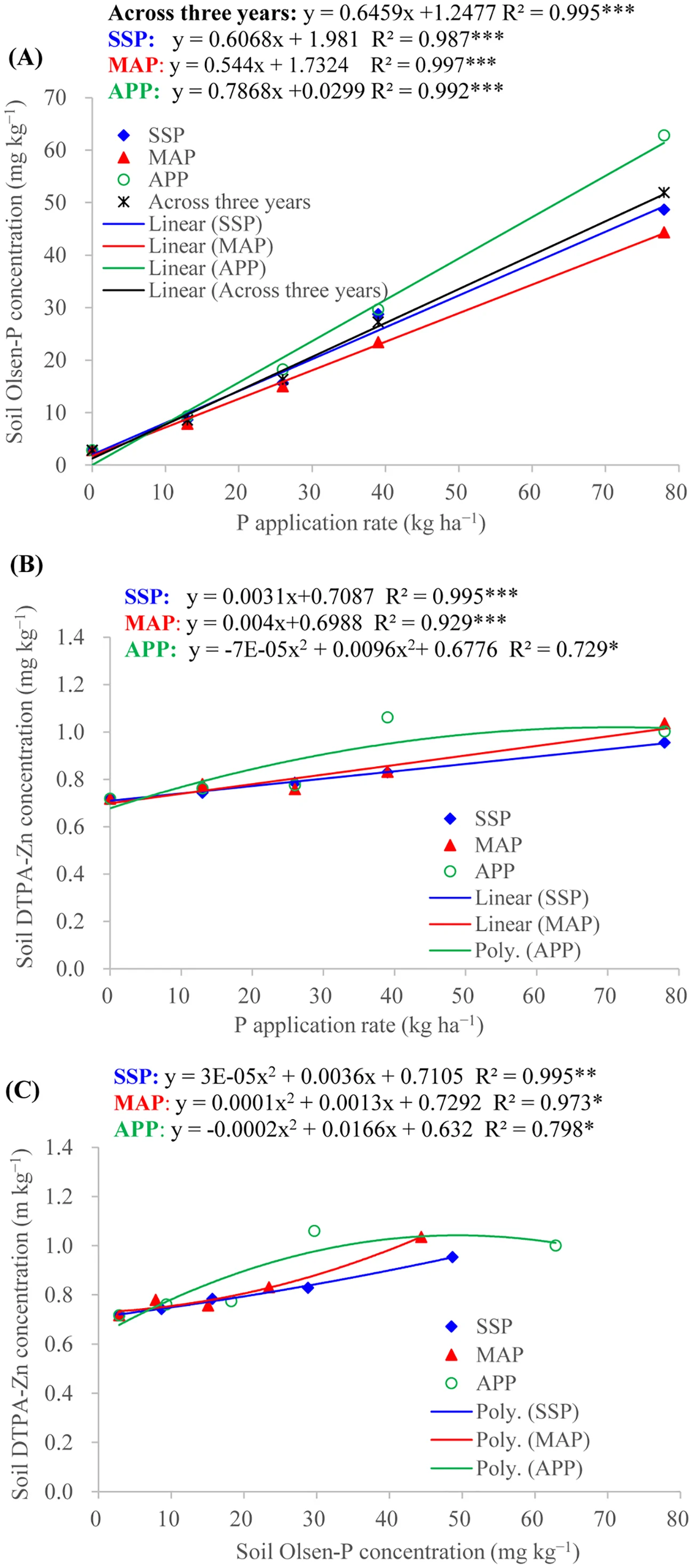
Response of soil Olsen-P concentrations **(A)** and soil DTPA-Zn concentrations **(B)** to the P application rates, and the relationship between soil DTPA-Zn concentrations and soil Olsen-P concentrations **(C)** with three fertilizer P sources during the three experimental years from 2020 to 2022. Fertilizer P sources include superphosphate (SSP), monoammonium phosphate (MAP) and ammonium polyphosphate (APP). Results are means of four replicates, each of which is the average of observations in the same plot across the three years. *significant at P < 0.05, **significant at P < 0.01, ***significant at P < 0.001.

The increase in soil Olsen-P concentration followed the order: MAP < SSP < APP (**Table 4**). Across the three years, the increase (slope of linear response curve) in Olsen-P per unit of applied P was 0.54 mg for MAP, 0.61 mg for SSP, and 0.79 mg for APP (**Figure 4A**). When averaged across fertilizer sources and years, the average increase was 0.65 mg (**Figure 4A**). Regression analysis further revealed that the DTPA-extractable Zn concentration increased linearly with P rate for SSP and MAP, and quadratically for APP (**Figure 4B**). DTPA-extractable Zn concentration exhibited a quadratic increase with soil Olsen-P concentration across all three P sources-SSP, MAP, and APP (**Figure 4C**).

### 3.6 Relationships between soil Olsen-P concentration and Zn concentration, Zn accumulation, and Zn harvest index

**Figure 5:**
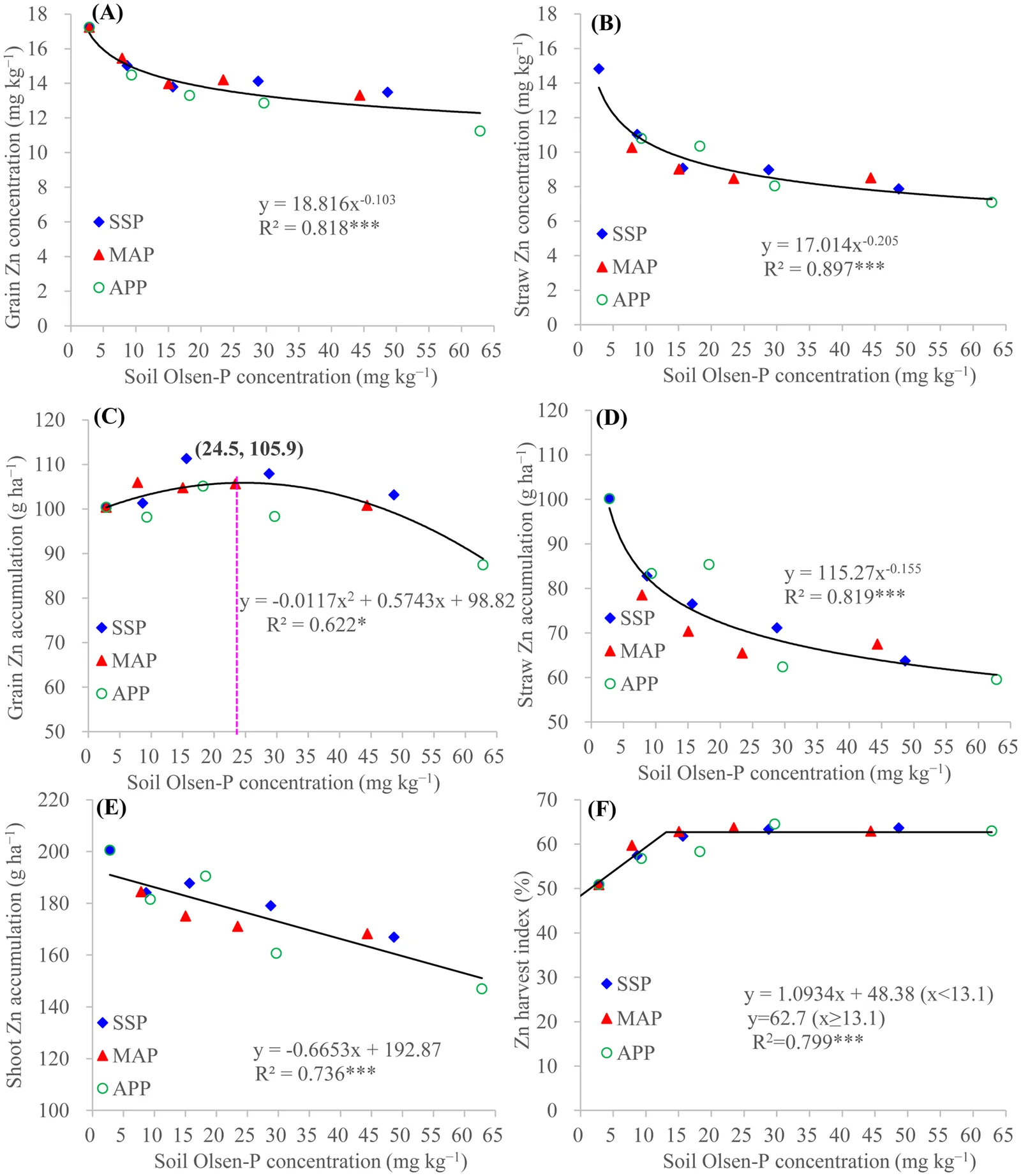
Response of grain Zn concentration **(A)**, straw Zn concentration **(B)**, grain Zn accumulation **(C)**, straw Zn accumulation **(D)**, aboveground Zn accumulation **(E)** and Zn harvest index **(F)** to soil Olsen-P concentrations at 0–20 cm depth with three fertilizer P sources during the three experimental years from 2020 to 2022. Fertilizer P sources include superphosphate (SSP), monoammonium phosphate (MAP) and ammonium polyphosphate (APP). Results are means of four replicates, each of which is the average of observations in the same plot across the three years. *significant at P <0.05, ***significant at P <0.001.

Across the three-year study, Zn concentration in grain and especially in straw, as well as Zn accumulation in straw, were well predicted by a negative power function of soil Olsen-P (**Figures 5A, B, D**). Grain Zn accumulation exhibited a quadratic decrease once soil Olsen-P concentration exceeded 24.5 mg kg^−1^ (**Figure 5C**). Overall, aboveground Zn accumulation declined linearly as soil Olsen-P increased (**Figure 5E**). A linear-plateau model indicated that Zn harvest index reached a plateau of 62.7% at Olsen-P threshold of 13.1 mg kg^−1^ across the three years (**Figure 5F**).

### 3.7 Principal component analysis of various parameters influenced by cropping years, P application rates and P sources

**Figure 6:**
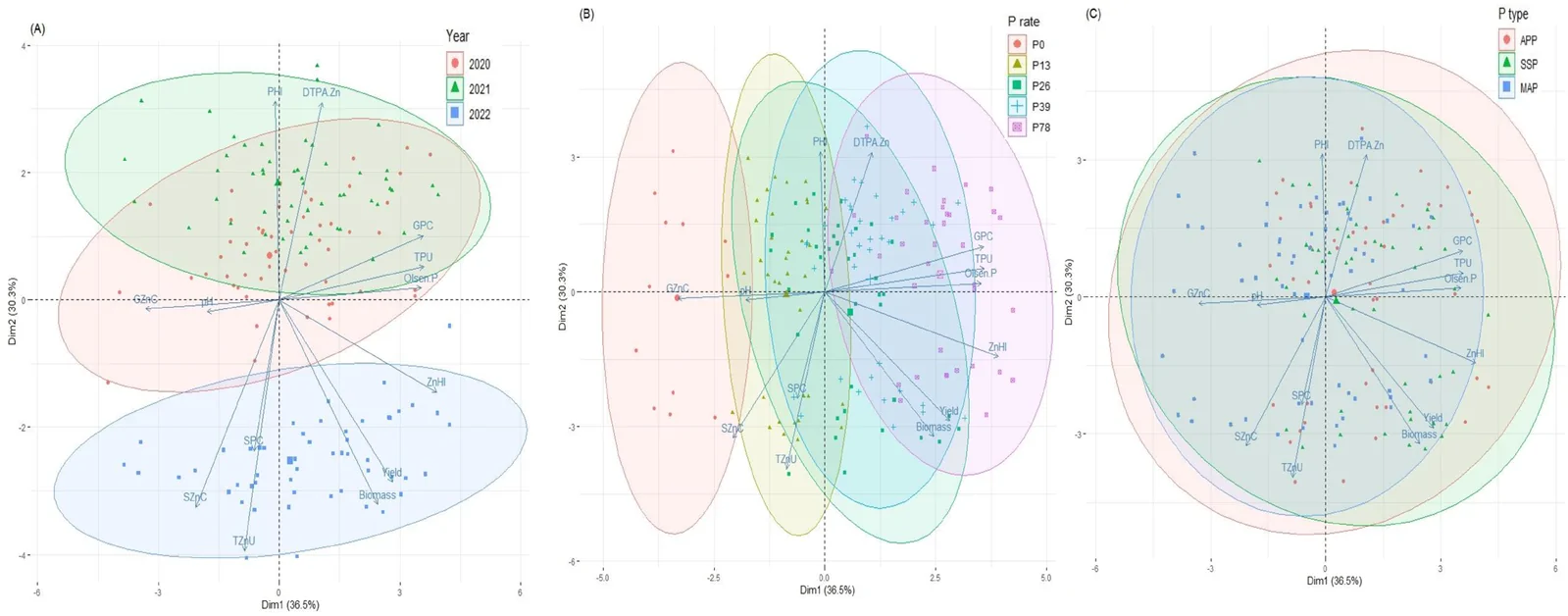
Principal component analysis (PCA) of the effects of different experimental years **(A)**, different P application rates **(B)** and fertilizer P sources **(C)** on various investigated parameters including grain yield, biomass accumulation, grain concentrations of Zn and P (recorded as GZnC and GPC), straw concentrations of Zn and P (recorded as SZnC and SPC), aboveground accumulation of Zn and P (recorded as TZnU and TPU), nutrient harvest index for Zn and P (recorded as ZnHI and PHI), soil pH values, soil Olsen-P concentrations (recorded as Olsen P) and soil DTPA-Zn concentrations (recorded as DTPA Zn). Fertilizer P sources include superphosphate (SSP), monoammonium phosphate (MAP) and ammonium polyphosphate (APP).

The principal component analysis (PCA) showed a clear view of the relationships among all measured parameters. It also illustrated data distribution across various treatments applied to the maize crop (**Figure 6**). The first two principal components accounted for 66.8% (PC1-36.5%, PC2-30.3%) of the total variance (**Figure 6A**). Data points were clustered by cropping years (**Figure 6A**) and P application rates (**Figure 6B**). In general, there was a distinct separation/difference in data distribution areas among the three cropping years, especially for 2022, and among the five P application rates, while differences among P sources were small.

**Figure 7:**
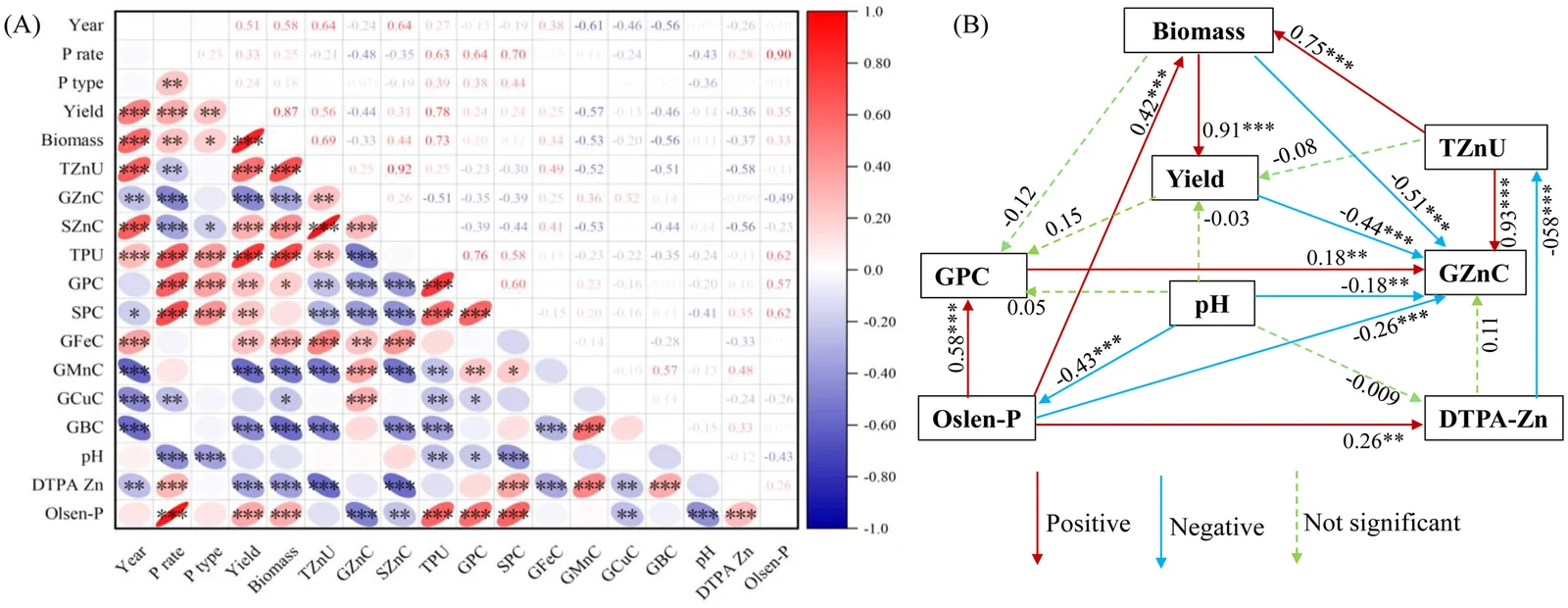
Corrplot **(A)** and partial least squares structural equation modeling (PLS-SEM) **(B)** estimating the relationships among soil nutrients, grain concentrations and crop nutrient uptake, and yield and biomass accumulation. Corrplot **(A)** representing correlation among measured grain yield, biomass accumulation, grain concentrations of Zn, Fe, Mn, Cu and P (recorded as GZnC, GFeC, GMnC, GCuC and GPC), straw concentrations of Zn and P (recorded as SZnC and SPC), aboveground accumulation of Zn and P (recorded as TZnU and TPU), soil pH values, soil Olsen-P concentrations and soil DTPA-Zn concentrations as affected by different experimental years, different P application rates and fertilizer P sources **(A)**. PLS-SEM **(B)** estimating the direct and indirect effects of soil properties (pH value, Olsen-P, and DTPA-Zn), maize growth parameters (Yield and Biomass) and nutrient uptake parameters (GPC, GZnC, TZnU). Path coefficients are illustrated using trajectory lines: blue indicates negative effects, red indicates positive effects, and dashed lines indicate no significance effects. Significance levels are indicated by *, **, and *** for P < 0.05, P < 0.01, and P < 0.001, respectively. The specific values of the SEM model fit indices were as follows: n = 156, SRMR = 0.039, NFI = 0.959.

Correlation analysis further revealed that maize grain Zn concentration was significantly positively correlated with grain Mn, Cu, Fe concentrations. Grain Zn concentration was negatively correlated with aboveground P uptake, soil Olsen-P concentration, grain yield, straw P concentration, grain P concentration, and biomass (**Figure 7A**). Structural equation model (SEM) further revealed the significantly negative direct effects of biomass, grain yield, soil Olsen-P, and soil pH value on grain Zn concentration, with path coefficients of -0.51, -0.44, -0.26 and -0.18, respectively (**Figure 7B**).

## 4 Discussion

### 4.1 Effect of fertilizer P rates and sources on maize yield

**Figure 8:**
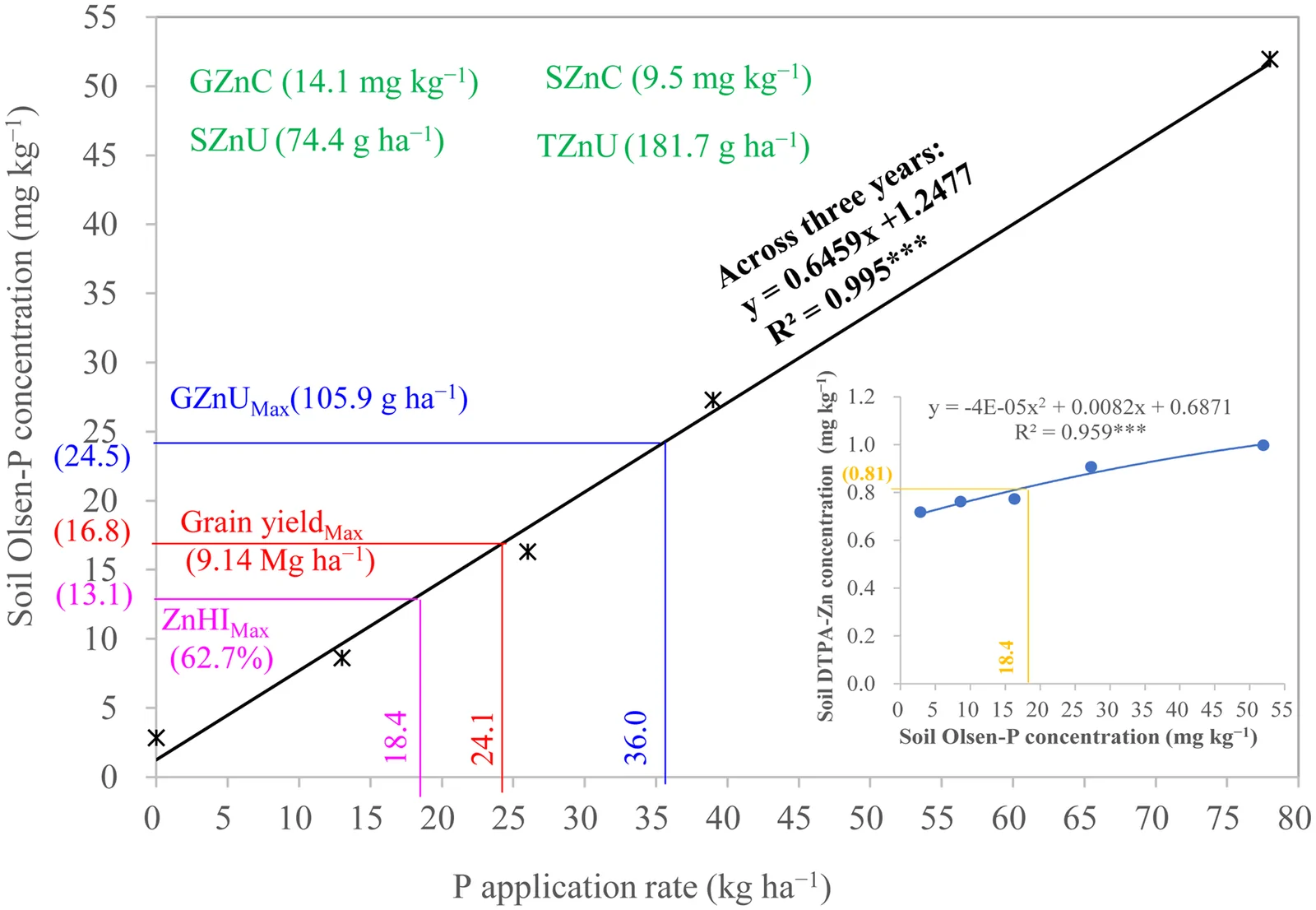
Required P application rates and soil Olsen-P concentrations at 0–20 cm depth to achieve the maximum grain yield (grain yield_Max_), grain Zn accumulation (GZnU_Max_) and Zn harvest index (ZnHI_Max_) across the three years. The values in parentheses indicate the grain yield_Max_, GZnU_Max_ and ZnHI_Max_ and the corresponding P application rates and Olsen-P concentrations. The values in parentheses for grain Zn concentration (GZnC), straw Zn concentration (SZnC), straw Zn accumulation (SZnU) and aboveground Zn accumulation (TZnU) were also estimated corresponding to the calculated highest grain yields based on the required P application rate of 24.1 kg ha^−1^ and soil Olsen-P concentration of 16.8 mg kg^−1^.

Consistent with previous studies (Bai et al., 2013; Zhang et al., 2017b), maize grain yield exhibited a clear linear-plateau response to increasing P fertilizer supply (**Figure 1**). In this study, maize yield increased with P application rates and reached a plateau of 9.14 Mg ha^–1^ at a soil Olsen-P concentration of 16.8 mg kg^–1^, corresponding to a P application rate of 24.1 kg ha^–1^ (**Figure 8**). This rate is close to the P26 treatment (26 kg P ha^−1^). These results indicate that applying fertilizer P at 26 kg ha^−1^ is an optimal rate for maize grown in saline-alkali soils. Our previous research demonstrated a similar pattern for wheat, with yield reaching a plateau of 6.7 Mg ha^–1^ at 18 kg P ha^−1^ under similar soil conditions (Liu et al., 2022). On a calcareous alluvial soil, maize yield increased with P rates and reached a plateau of 9.67 Mg ha^–1^ at an Olsen-P level of 11.8 mg kg^–1^, corresponding to P application rate of 19.5 kg ha^–1^ (Zhang et al., 2017b). Together, these findings suggest that the critical soil P level needed to achieve the yield plateau differs across regions due to differences in crop species, soil type, pH, and soil organic matter content (Bai et al., 2013). Under the optimized application rate (P26 treatment), average yield over three years was highest for SSP, followed by APP while MAP resulted in the lowest yields (**Figure 1B**). This indicates that SSP is the most effective P type for enhancing maize yield in coastal saline soils, which aligns with our previous studies (Liu et al., 2022; Wu et al., 2025). This could be explained by a number of factors. First, SSP contains higher exchangeable Ca^2+^ and effectively replaces high Na^+^ from soil colloids, thereby enhancing soil cation exchange capacity and improving soil structure and reducing particle dispersion (Liu et al., 2022; Rengasamy and Marchuk, 2011). Second, SSP facilitates uptake of P, K, Ca and Mg in maize plants, leading to increased ratios of Ca/Na, K/Na, and Mg/Na (Wu et al., 2025). Such improvements further promote ionic balance and alleviate Na^+^ toxicity, ultimately resulted in increased crop biomass and grain yield. Third, the sulfur, silicon and Mg provided by SSP promote maize plant growth by enhancing protein synthesis, promoting photosynthetic activity, and improving stress tolerance (Sharma et al., 2024).

### 4.2 Effect of P rates on Zn concentration and accumulation in grain and straw

Compared to no P input, increasing P supply significantly reduced grain Zn concentration by 13.1–26.4% (average 20.1%), and straw Zn concentration even more pronounced by 27.9–47.2% (average 38.5%). A similar pattern was reported in a meta-analysis, which showed greater reduction in straw Zn (28.2%) compared to grain Zn (20.2%) under higher P supply (Zhang et al., 2021). Consequently, both the grain-to-straw Zn ratio and ZnHI increased with higher P rates (**Table 2**; **Figure 3A**; **Supplementary Figure 1**). These authors also reported that P application had no significant effects on straw-to-grain Zn ratios or root-to-aboveground Zn ratios in cereal crops such as maize, wheat and rice (Zhang et al., 2021). These results indicate that root-to-aboveground Zn translocation and straw-to-grain Zn remobilization were not the main limiting factors for grain Zn decline associated with higher P application rates in saline-alkali soils. Soil DTPA-Zn increased linearly or quadratically as P application rates increased (**Figure 4**), indicating soil Zn solubility was not be the primary factor contributing to P-Zn antagonism under saline-alkali conditions. Numerous studies have shown that grain Zn concentration reduced at higher soil Olsen-P levels, primarily due to a dilution effect associated with enhanced yield and reduced mycorrhizal colonization (Hui et al., 2019; Ova et al., 2015; Zhang et al., 2017a). In our study, aboveground biomass and grain yield increased by 25.7% and 36.3%, respectively, while aboveground Zn accumulation decreased by 19.8% as P rates increased from P0 to P26 (**Table 1**; **Figure 2**). The observed decline was mainly due to a “dilution effect” resulted from increased biomass production. Hui et al. (2019) also showed the decrease in wheat grain Zn concentration was mainly ascribed to yield dilution effects, because the soil available Zn concentration and root AMF colonization was not changed with increasing P application at lower available soil P levels. When fertilizer P supply further increased from P26 to P78, aboveground biomass or grain yield no longer changed, but aboveground Zn concentration and accumulation still declined by 12.4% and 12.9%, respectively. The decline in aboveground Zn accumulation is likely attributable to decreased root colonization by AMF, rather than a dilution effect. Ova et al. (2015) reported that the negative impacts of increasing P supply on root Zn uptake in wheat depend strongly on mycorrhiza, may disappear when mycorrhizal associations are absent. The mycorrhizal pathway of Zn uptake contributed up to 24.3% of aboveground Zn uptake in wheat (Coccina et al., 2019). However, the efficiency of Zn acquisition through mycorrhizal symbiosis can be significantly diminished by P supply, as it substantially impairs root AM colonization (Lehmann et al., 2014; Zhang et al., 2017a). Similarly, P-induced decreases in Zn concentration, Zn uptake, and Zn acquisition efficiency were more pronounced in mycorrhizal crops such as maize and soybean than in non-mycorrhizal oilseed rape (Yu et al., 2024). Furthermore, the disruption of the mycorrhizal pathway through the mutation of ZmPht1;6 led to impaired growth in maize plants and a 1.6-fold reduction in aboveground Zn concentration (Yu et al., 2020). This decline was attributed to a significant decrease in arbuscular mycorrhizal colonization and hyphal length density (HLD). Additionally, the expression levels of Zn transporter genes, specifically ZmZIP3, ZmZTP29, and ZmIRT1 were notably downregulated by P application under both field conditions and phytochamber experiments. The high P treatment also reduced root hyphal Zn uptake, potentially through suppressing the expression of fungal RiZRT1 and RiZnT1 genes (Yu et al., 2020). These findings suggest that the adverse effects of P supply on Zn uptake efficiency may occur via both AM symbiosis and hyphae-mediated pathways for Zn acquisition on saline-alkali soils. Further investigation into the underlying mechanisms is needed. New evidence also shows that Zn efficient wheat cultivars can partially overcome P-Zn antagonism by recruiting more beneficial rhizobacteria, maintaining AMF colonization, and supporting stronger root growth (Yang et al., 2025). In this study, the observed decline in maize Zn concentration and accumulation suggest that the maize cultivar used may be not be highly efficient at Zn absorption under saline-alkali conditions. Additionally, aboveground accumulation of Fe, Mn, Cu, and B increased significantly as the P supply increased from P0 to P13 or P26, but further P supply did not affect the aboveground accumulation of these nutrients. Conversely, a significant decrease in aboveground Zn accumulation of maize with increasing levels of P supply (Zhang et al., 2017b). These findings suggest that Zn deficiency in saline-alkali soil, especially under excessive P application, represents a critical limiting factor for enhancing both maize yield and grain Zn concentration. Furthermore, the pronounced interannual variability in rainfall during 2020–2022 likely induced contrasting responses between soil and plant compartments. In 2022, intense precipitation events triggered substantial leaching of plant-available nutrients, resulting in reduced DTPA-extractable concentrations of Zn, Fe, Mn, and Cu in the soil. Concurrently, enhanced transpiration rates and greater biomass accumulation improved nutrient uptake capacity, particularly for Zn and Fe, thereby partially compensating for the soil-based nutrient deficit.

### 4.3 Effect of P rate and source on crop growth, yield, and Zn nutrition

Recent work has shown that applying DAP to wheat grown in lime concretion black soil, and applying triple superphosphate (TSP) or APP to fluvo-aquic soil, can improve grain Zn enrichment and ensure high P use efficiency under pot experimental conditions (Zhang et al., 2024). Our previous study also found that SSP significantly increased wheat yield by 14%, while increasing aboveground P accumulation by 14% and 13% compared to MAP and APP, respectively, under the optimal P application rate on coastal saline soil (Liu et al., 2022). In contrast, no effect of P sources on grain yield or aboveground P uptake of maize grown on calcareous soil (Wang et al., 2024). Gao et al. (2020) reported that APP, especially with high polymerization degree, significantly increased soil available P, Zn, Fe, and Mn, which in turn improved their uptake by maize grown on calcareous soil under greenhouse conditions. In this study, P fertilizer sources had no significant effects on straw Zn concentration and soil DTPA-Zn. However, although APP increased soil available Zn at appropriate P application rates, it also reduced grain Zn concentration (**Table 1**; **Figure 4**). This reduction may be due to the stronger ability of APP to increase soil Olsen-P (**Figure 4A**), which likely inhibited AMF colonization, reduced root Zn uptake, and ultimately lowered grain Zn concentrations in saline-alkali soils. Previous studies have demonstrated that soil application of Zn fertilizer is crucial for improving maize yield while simultaneously promoting soil microbial activity and improving the bacterial community (Liu et al., 2020). Foliar Zn spraying has also proven effective for improving grain Zn of maize in our previous study (Xue et al., 2023). Our recent findings show that applying SSP at an optimized P rate could successfully improve soil-crop system salt ion equilibrium to increase agricultural ecosystems productivity (Wu et al., 2025). Therefore, under saline-alkali conditions, applying SSP at an optimized rate (26 kg P ha^–1^), combined with either soil application or foliar spraying of Zn fertilizer, may represents a promising strategy for achieving high maize yields and elevating grain Zn concentration.

### 4.4 Effect of P rates and types on bioavailability of micronutrients in grain and straw

Consistent with previous studies (Stein, 2010; Zhao et al., 2020), high P application increased soil Olsen-P level and substantially reduced grain concentrations of Zn, Fe, and Cu, but not Mn, and concurrently enhanced grain P concentration. Phytic acid is the predominant P storage compound in seeds, accounting for up to 80% of the total P content. However, PA impairs biological P utilization by chelating nutritionally essential minerals, such as Zn and Fe (Febles et al., 2002). As a result, the molar ratios of P to Zn, Fe, Mn, and Cu increased significantly, indicating lower bioavailability of these essential micronutrients (Zhang et al., 2017b). Such changes adversely affect the absorption of these vital micronutrients by the human body (Cakmak, 2008; Imran and Rehim, 2016). In China, maize is typically harvested as either grain or silage depending on market demand. The large decline in straw concentrations of Zn and Mn, alongside an increase in P, suggests a similar reduction in the bioavailability of Zn, Fe, Mn, and Cu in maize straw (**Table 3**). This trend indicates a decline in the bioavailability of essential micronutrients, particularly Zn for human consumption and animal feed.

### 4.5 Grain yield and Zn concentration and accumulation in response to P rate and soil Olsen-P

The highest grain yield of 9.14 Mg ha^–1^ was achieved at a soil Olsen-P concentration of 16.8 mg kg^–1^, corresponding to a P application rate of 24.1 kg P ha^–1^. Maximum grain Zn accumulation (105.9 g ha^–1^) was achieved at a higher soil Olsen-P concentration of 24.5 mg kg^–1^ with a P rate of 36.0 kg P ha^–1^. Furthermore, the highest Zn harvest index (62.7%) was obtained at a soil Olsen-P concentration of 13.1 mg kg^–1^ with a P application rate of 18.4 kg ha^–1^. However, both P rate and soil Olsen-P had strong negative effects on Zn concentrations in grains and particularly straw (**Figures 2**, **5**). At the P rate of 24.1 kg P ha^−1^ or soil Olsen-P of 16.8 mg kg^–1^, measured Zn concentrations were approximately 14.1 mg kg^−1^ in grains and 9.5 mg kg^−1^ in straw, while the DTPA-extractable Zn concentration was recorded at around 0.81 mg kg^−1^. Consequently, Zn accumulation in straw and aboveground plants remained low, at 74.4 g ha^−1^ and 181.7 g ha^−1^, respectively (**Figure 8**).

It is plausible that enhanced grain yield and biomass production contribute to greater internal Zn utilization efficiency, shown by lower aboveground Zn accumulation per unit grain yield, and enhanced Zn remobilization to grain, shown by increased ZnHI. This enhancement occurs at the cost of reduced Zn concentrations in both grain and especially straw (Xue et al., 2014). Similarly higher plant internal Zn utilization efficiency has been previously reported for maize compared to legumes and rice (Fageria et al., 2008).

## 5 Conclusion

Our three-year field experiment demonstrated that applying SSP at an optimized rate (26 kg P ha^–1^) improved maize yield in saline-alkali soil. However, grain Zn concentration significantly decreased from 17.2 to 12.7 mg kg^–1^ with the increasing P rate from P0 to P78. Among the three P sources, SSP and MAP resulted in an increase of 8.7% and 9.7% in grain Zn concentration compared to APP. These concentrations remain substantially below the target values of 38 mg kg^−1^ for maize grain Zn (Bouis et al., 2011). Given that saline-alkali soils inherently pose a high risk for Zn deficiency, combined with evidence that excessive P application further inhibits root Zn uptake and its translocation to grains, it is crucial to implement effective strategies under these conditions. Structural equation modeling revealed that aboveground biomass, grain yield, soil Olsen-P, and soil pH negatively affected grain Zn concentration and should be more extensively considered in the production and nutritional quality of maize grain Consequently, applying SSP at an optimized rate (26 kg P ha^–1^), combined with either soil application or foliar spraying of Zn fertilizer, represents a promising strategy for achieving high maize yields and elevating grain Zn concentration in saline-alkali soils in future.

## Data Availability

The original contributions presented in the study are included in the article/**Supplementary Material**. Further inquiries can be directed to the corresponding author.
