# Supplementary material for: Optimizing phosphate fertilizer rate and sources for enhancing yield and grain zinc of maize under saline conditions
Source: Front Plant Sci. 2026 Sep 10;17:1937429. doi: 10.3389/fpls.2026.1937429 (PMC13601314; doi:10.3389/fpls.2026.1937429)
Supplement: Supplementary file 1 [file DataSheet1.docx]

**Fig. S1** The rations of grain to straw Zn concentrations as affected by different P application rates across these three years and different fertilizer P sources

**Table S1** P concentrations in grain and straw of maize as affected by P rates, P sources, and cropping years. Fertilizer P sources include superphosphate (SSP), monoammonium phosphate (MAP) and ammonium polyphosphate (APP). Values represent the mean of four replicates. Within a column, different letters denote significant difference at *P* < 0.05. ns indicates not significant at *P* < 0.05. GPC, grain P concentration; SPC, straw P concentration; GPU, grain P accumulation; SPU, straw P accumulation, TPU, aboveground P accumulation.

| Treatment | GPC | SPC | GPU | SPU | TPU |
| --- | --- | --- | --- | --- | --- |
|  | (g kg^–1^) | (g kg^–1^) | (kg ha^–1^) | (kg ha^–1^) | (kg ha^–1^) |
| P rate (R) |  |  |  |  |  |
| P0 | 1.55d | 0.35e | 9.0d | 2.3d | 11.2d |
| P13 | 1.96c | 0.42d | 13.3c | 3.1c | 16.4c |
| P26 | 2.03bc | 0.47c | 16.0b | 3.6b | 19.7b |
| P39 | 2.09b | 0.51b | 15.9b | 3.7b | 19.6b |
| P78 | 2.37a | 0.55a | 18.2a | 4.2a | 22.5a |
| P source (S) |  |  |  |  |  |
| SSP | 2.22a | 0.53a | 16.9a | 4.0a | 20.0a |
| MAP | 2.00c | 0.45c | 14.7c | 3.3c | 18.1c |
| APP | 2.12b | 0.48b | 16.0b | 3.7b | 19.6b |
| Year (Y) |  |  |  |  |  |
| 2020 | 2.13a | 0.49a | 15.1b | 3.4b | 18.5b |
| 2021 | 2.06b | 0.48a | 13.7c | 3.2c | 16.9c |
| 2022 | 2.03b | 0.46b | 17.2a | 4.1a | 21.3a |
| Source of variation | |  |  |  |  |
| Y | 0.0038 | <0.0001 | <0.0001 | <0.0001 | <0.0001 |
| R | <0.0001 | <0.0001 | <0.0001 | <0.0001 | <0.0001 |
| S | <0.0001 | 0.0012 | <0.0001 | <0.0001 | <0.0001 |
| Y*R | ns | 0.0424 | ns | 0.0283 | ns |
| Y*S | 0.0003 | <0.0001 | 0.0021 | 0.0014 | <0.0001 |
| R*S | 0.0004 | ns | 0.0006 | ns | 0.0002 |
| Y*R*S | ns | ns | ns | ns | ns |
